# Fumonisin B_1_ Production by *Fusarium* Species and Mycotoxigenic Effect on Larval Zebrafish

**DOI:** 10.21315/tlsr2020.31.3.7

**Published:** 2020-10-15

**Authors:** Najihah Azman, Nur Ain Izzati Mohd Zainudin, Wan Norhamidah Wan Ibrahim

**Affiliations:** Department of Biology, Faculty of Science, Universiti Putra Malaysia, 43400 Serdang, Selangor, Malaysia

**Keywords:** Fumonisin B_1_, Toxic, *Fusarium*, Malaysia, Ultra-Fast Liquid Chromatography, Fumonisin B_1_, Toksik, *Fusarium*, Malaysia, Kromatografi Cecair Ultra-Cepat

## Abstract

Fumonisin B_1_ (FB_1_) is a common mycotoxin produced by *Fusarium* species particularly *F. proliferatum* and *F. verticillioides*. The toxin produced can cause adverse effects on humans and animals. The objectives of this study were to detect the production of FB_1_ based on the amplification of *FUM1* gene, to quantify FB_1_ produced by the isolates using Ultra-fast Liquid Chromatography (UFLC) analysis, to examine the embryotoxicity effect of FB_1_ and to determine EC_50_ toward the larvae of zebrafish (*Danio rerio*). Fifty isolates of *Fusarium* species were isolated from different hosts throughout Malaysia. Successful amplification of the *FUM1* gene showed the presence of this gene (800 bp) in the genome of 48 out of 50 isolates. The highest level of FB_1_ produced by *F. proliferatum* isolate B2433 was 6677.32 ppm meanwhile *F. verticillioides* isolate J1363 was 954.01 ppm. From the assessment of embryotoxicity test of FB_1_ on larvae of zebrafish, five concentrations of FB_1_ (0.43 ppm, 0.58 ppm, 0.72 ppm, 0.87 ppm and 1.00 ppm) were tested. Morphological changes of the FB_1_ exposed-larvae were observed at 24 to 168 hpf. The mortality rate and abnormality of zebrafish larvae were significantly increased at 144 hpf exposure. Meanwhile, the spontaneous tail coiling showed a significant difference. There were no significant differences in the heartbeat rate. As a conclusion, the presence of *FUM1* in every isolate can be detected by *FUM1* gene analysis and both of the species produced different concentrations of FB_1_. This is the first report of FB_1_ produced by *Fusarium* species gave a significant effect on zebrafish development.

## Abstract (ms)

Fumonisin B_1_ (FB_1_) ialah mikotoksin biasa yang dihasilkan oleh spesies *Fusarium*, terutamanya *F. proliferatum* and *F. verticillioides*. Toksin yang dihasilkan boleh menyebabkan kesan yang buruk terhadap manusia dan haiwan. Objektif kajian ini adalah untuk mengesan penghasilan FB_1_ berdasarkan amplifikasi gen *FUM1*, mengukur kuantiti FB_1_ yang dihasilkan oleh pencilan menggunakan analisis Kromatografi Cecair Ultra-Cepat (UFLC), memeriksa kesan embriotoksik FB_1_ dan mengenal pasti EC_50_ terhadap ikan zebra (*Danio rerio*). Lima puluh spesies *Fusarium* diasingkan daripada perumah berlainan di seluruh Malaysia. Amplifikasi gen *FUM1* pada 48 daripada 50 pencilan berjaya menunjukkan kehadiran gen ini (800 bp). Pencilan *F. proliferatum* B2433 menghasilkan FB_1_ pada aras tertinggi sebanyak 6677.32 bpm manakala pencilan *F. verticillioides* J1363 sebanyak 954.01 ppm. Berdasarkan penilaian ujian embriotoksik FB_1_ ke atas larva ikan zebra, lima kepekatan FB_1_ (0.43 ppm, 0.58 ppm, 0.72 ppm, 0.87 ppm dan 1.00 ppm) telah diuji. Perubahan morfologi larva yang terdedah kepada FB_1_ diperhatikan pada 24 hingga 168 hpf. Kadar kematian dan ketidaknormalan larva ikan zebra meningkat pada pendedahan 144 hpf. Sementara itu, penggulungan ekor secara spontan menunjukkan perbezaan yang bererti. Tiada perbezaan yang bererti terhadap kadar degupan jantung. Kesimpulannya, kehadiran *FUM1* pada setiap pencilan boleh dikesan dengan analisis gen *FUM1* dan kedua-dua spesies menghasilkan kepekatan FB_1_ yang berbeza. Hasil kajian ini merupakan laporan pertama FB_1_ yang dihasilkan oleh spesies *Fusarium* yang memberikan kesan yang bererti ke atas perkembangan ikan zebra.

**Highlights**

- *Fusarium proliferatum* and *Fusarium verticillioides* isolates can produce a high level of FB_1_.
- Embryotoxicity test of FB_1_ on zebrafish showing significant effects on the mortality rate and morphological abnormalities.
- The effects of FB_1_ exposure toward zebrafish larvae can be observed as early as 24 hpf and EC_50_ of FB_1_ towards zebrafish was 0.569 ppm.

## INTRODUCTION

Mycotoxins contamination occurs frequently in the area with a hot and humid climate where it is the most suitable condition for the growth of the fungi. Factors that influence the contamination of mycotoxins in food and feedstuff are related to a favourable environmental condition such as unsterile storage area (Zain 2011). One of the most common mycotoxins is Fumonisin B_1_ (FB_1_) produced by *Fusarium* species. At least 10 different species of *Fusarium* can produce this toxin, however, *Fusarium proliferatum* and *F. verticillioides* are the most common fungi associated with crops (WHO/FAO 2001). Apart from crops, both species can also be isolated from varieties of plants and are spread by rain and other abiotic factors.

Production of FB_1_ by *Fusarium* species is dependent on a biosynthetic gene cluster *FUM*, which consists of *FUM1* to *FUM19* where *FUM1* is a gene that responsible for the production of FB_1_ (Glenn *et al*. 2008). FB_1_ is classified into Group 2B carcinogens by the International Agency for Research on Cancer (IARC). FB_1_ is toxic and causing leukoencephalomalacia and it has been reported to be associated with human oesophageal cancer and birth defects (Marasas 2001). The occurrence of mycotoxin contamination has become a great concern because it is often associated with acute and chronic diseases (Logrieco *et al*. 2002). Besides, it also can compromise the safety of food and feed supplies in humans and animals.

As mycotoxins contamination in plant-based products become serious problems, it is important to conduct this study in order to detect and prevent human and animal exposure to toxic substances. To detect accurately the fumonisin production in *F. proliferatum* and *F. verticillioides*, a diagnostic method based on polymerase chain reaction (PCR) is the most suitable and rapid detection by using specific *FUM1* gene pairs as it detects the genes responsible to produce fumonisin. Meanwhile in order to examine the effect of FB_1_ towards human and animal health uses of zebrafish (*Danio rerio*) as a model organism is important as it has been reported that 70% of human genes have zebrafish orthologues (Howe *et al*. 2013). Since currently there are no reports on the exposure of FB_1_ on zebrafish, from this study, it can provide more information on the effect of FB_1_ thus can relate to human health and development. In addition, there were different types of mycotoxins that have been tested on zebrafish embryo such as ochratoxin, T-2 toxin and zeralenone. Wu *et al*. (2016) have reported that zebrafish embryo that were exposed with ochratoxin have abnormal heart looping and small heart chambers. The toxin also has disrupts the renal morphology of the embryonic zebrafish. Meanwhile, another mycotoxin exposure on zebrafish embryo was T-2 toxin where higher concentration of T-2 toxin can significantly increase the mortality rate and also caused the malformations on tails and cardiovascular defects (Yuan *et al*. 2014). Zeralenone exposure on zebrafish showing defects on eye development and also upward curvature of the body axis (Bakos *et al*. 2013).

The objectives of this study were: (i) to detect the production of FB_1_ based on the amplification of *FUM1* gene and to quantify FB_1_ produced by the isolates using Ultra-fast Liquid Chromatography (UFLC) analysis, and (ii) to examine the embryotoxicity effect of FB_1_ on the larvae of zebrafish (*D. rerio*) and (iii) to determine EC_50_ of FB_1_ towards zebrafish.

## MATERIALS AND METHODS

### Fungal Source and Preservation

Fifty isolates of *Fusarium proliferatum* and *F. verticillioides* were obtained from Mycology Laboratory, Department of Biology, Universiti Putra Malaysia and Plant Pathology Laboratory, School of Biological Sciences, Universiti Sains Malaysia. The isolates were previously isolated from different hosts and cultured on potato dextrose agar (PDA) and incubated at 27 ± 2°C for five days. All pure cultures were transferred onto Spezieller Nahrstoffarmer Agar (SNA) and sterile filter paper for preservation purpose. The culture stocks were kept in −20°C for further use. Species identification of the isolates was confirmed by using species-specific sequence analysis of ProF1/ProR1 and VertF1/VertR1 (Najihah *et al*. 2017).

### DNA Extraction and *FUM1* Gene Amplification

The isolates were cultured on PDA and incubated at 27 ± 2°C for 7 days and genomic DNA was extracted by using Qiagen DNeasy® UltraClean® Microbial Kit according to the protocols provided by the manufacturer. *FUM1* gene detection has been carried out by using primers FUMF1 (5′-GACACCTCCTTCTTCTC-3′) and FUMR1 (5′-GTGCCGGTTCCGTGTGCTTC-3′) (Proctor *et al*. 1999). Standard PCR master mix GoTaq® Flexi DNA Polymerase Promega Corporation for a total of 20 μL was consisted of green buffer, 2 mM dNTP, 25 mM MgCl_2_, 10 mM of each forward and reverse primer respectively, 1 unit *Taq* polymerase, nuclease-free water and 20 ng DNA. The amplifications were performed by using Biometra TProfessional Basic Thermocyclers. The PCR condition was performed as follows; initial denaturation at 95°C for 5 min, 40 cycles of denaturation at 95°C for 10 s, annealing at 60°C for 10 s, extension at 72°C for 30 s and final extension at 72°C for 5 min (Proctor *et al*. 1999). Gel electrophoresis was performed by using 1.5% agarose gel immersed in 1X Tris Borate-acid EDTA (TBE) buffer amended with FloroSafe DNA stain (1^st^ Base Asia). Approximately 5 μL for each DNA ladder 100 bp (Thermo Scientific) and PCR products were loaded and electrophoresed for 35 min at 90 V.

### Inoculation and Extraction of FB_1_

All 50 isolates of *F. proliferatum* and *F. verticillioides* were cultured on PDA and incubated at 27°C ± 2°C for 7 days. The spores were dislodged by using 10 mL of sterile distilled water and filtered by using gauze. The final concentration of conidial suspension was adjusted to 1 × 10^5^ conidia mL^−1^ (Nur Ain Izzati *et al*. 2008). Inoculums were prepared by adding 85 g of cornmeal into 100 mL of distilled water and autoclaved at 121°C for 20 min. Another 5 mL of sterile distilled water was added and re-autoclaved for 30 min at 121°C. About 1 mL of conidial suspension was added into the sterile cornmeal and shaken for three continuous days to allow even distribution meanwhile for control cornmeal; 1 mL of sterile distilled water was added. The cultures were done in triplicates and then incubated at 28°C ± 2°C for 28 days (Nur Ain Izzati & Nithiyaa 2015). After the incubation period, 25 g of colonised cornmeal was ground in 100 mL of methanol: water (3:1; v/v). The extract then was filtered through Whatman No. 1 filter paper. The extract was then dried using rotary evaporator at 60°C, re-dissolved in 1 mL methanol and kept in −20°C freezer before analysed (Shephard 1998).

### Quantification of FB_1_ by using UFLC

Ortho-Phthaldialdehyde (OPA) reagent was freshly prepared before used by adding 5 mg of OPA (≥ 97% HPLC, Sigma-Aldrich, St Louis, MO, USA) with 125 μL of 95% ethanol, 4.9 mL of 0.1 M phosphate buffered-saline (PBS) pH 7.4 and 10 μL of β-mercaptoethanol (Go *et al*. 2008). OPA reagent, mobile phase and standard were filtered using polytetrafluoroethylene (PTFE) membrane before used. Stock solutions of FB_1_ standard was prepared (1 mg mL^−1^ in acetonitrile-H2O (1:1)) and aliquots used to prepare an evaporated working solution containing the FB_1_ at individual concentrations of 5 μg mL^−1^. For compiling matrix-matched calibration curves, five different concentrations of FB_1_ standard (2, 4, 6, 8 and 10 ppm) were prepared.

The sample was quantified by derivatised the extract by adding 50 μL of the sample with 200 μL of OPA reagent. Quantification of FB_1_ emitted by the *Fusarium* isolates was conducted using a Shimadzu (Kyoto, Japan) UFLC equipped with two LC-20AD pumps, a SIL-20A autosampler, a Shimadzu RF-10A XL fluorescence detector (at 335 nm excitation wavelength and 440 nm emission) and a Shimadzu SPD-20AV UV/VIS detector. A reversed-phase LC column, Thermo Scientific, Hypersil GOLD, C18, 250 mm × 4.6 mm i.d with a particle size of 5 μm was used. Approximately 20 μL of derivatised samples and standard were injected into the column using deionised water as a mobile phase A and 80% methanol: 0.1 M sodium dihydrogen phosphate (78:22/v:v) as a mobile phase B with flow rate of 0.8 mL min^−1^ (mobile phase A) and 0.2 mL min^−1^ (mobile phase B) for 10 min. Concentrations of FB_1_ were determined by comparing the formed peak observed using a fluorescent detector with the standard curve (Shephard 1998).

### Embryotoxicity Effect of FB_1_ in Zebrafish Larvae

Adult wild type zebrafish (5–8 months old with size 3 cm–5 cm) were provided by Aquatic Laboratory, Department of Biology, Universiti Putra Malaysia. All the adult zebrafish were maintained in recirculating freshwater aquarium system at 25°C–27°C on a ratio of 14 h light: 10 h dark of light cycle to induce a reproductive cycle of the fishes (Matthews *et al*. 2002). At 4 h post-fertilisation (hpf), fertilised eggs that reached the gastrulation stage were chosen for FB_1_ exposure. Standard FB_1_ from *Fusarium moniliforme* (≥ 98%, Sigma-Aldrich, St Louis, MO, USA) was diluted to final concentration (0.43, 0.58, 0.72, 0.87 and 1.00 ppm) in embryonic media solution. Three replicates of 96 well plates were used. One embryo for each well (*n* = 48 embryos per exposure group) was exposed to FB_1_ and control that contained embryonic media in a semi-static condition and incubated at 28 ± 2°C.

Percentage of mortality rate and morphological deformities were observed at 24, 48, 72, 97, 120, 144 and 168 hpf by using a dissecting microscope model Motic SMZ-161 (Motic Instrument, China). Tail coiling and heartbeat rate were counted within 1 min at 24 hpf and 48 hpf, respectively for every larva according to Organisation for Economic Co-operation and Development (OECD) guideline (OECD 2012), in which the heartbeat is visible and can be observed clearly at 48 hpf (Kalueff *et al*. 2013). Pericardial edema, yolk sac edema, non-inflated swim bladder and spinal curvature were determined using the guidelines of Scherz *et al*. (2008), Chakraborty *et al*. (2009), Hill *et al*. (2009) and Tsai *et al*. (2011), respectively.

Effective concentration (EC_50_) was calculated based on the concentration-response curve. The concentrations were converted to log value, and then a graph was plotted response against the log of the FB_1_ concentration. The EC50 was converted by taking the anti-log. The statistical analyses were conducted using SPSS version 21.0. One-way analysis of variance (ANOVA) followed by Duncan test *p* < 0.05 was set as a criterion for statistical significance to determine the differences between all concentrations to control treatments. Data were represented as mean ± standard deviation.

## RESULTS

### *FUM1* Gene Detection

All 48 isolates showed a positive result of *FUM1* gene where 800 bp of amplicons were amplified and clearly visible. Twenty-one isolates were *F. verticillioides* and 27 isolates were *F. proliferatum*. Two isolates of *F. proliferatum*, B68, and B92 were unable to amplify the *FUM1* gene.

### Quantification of FB_1_ Production using UFLC Analysis

From the standard curve, concentrations of the samples can be calculated based on the gradient formula (*y* = 5287.2*x* + 31641) from standard curve and peak area of the chromatogram. Table 1 shows the concentration of FB_1_ produced by *F. verticillioides* and *F. proliferatum*. Eighteen isolates of *F. verticillioides* produced FB_1_ meanwhile three isolates, B106, B1371 and 7706 did not produce FB_1_. The highest concentration of FB_1_ was produced by isolate J1363 with the concentration of 954.01 ppm meanwhile the lowest concentration was isolate 7696 with a concentration of 12.26 ppm. For *F. proliferatum*, 26 isolates produced FB_1_ meanwhile three isolates B68, B92 and P202 gave no result. The highest FB_1_ concentration was produced by isolate B2433 with concentration 6677.32 ppm meanwhile the lowest FB_1_ concentration was produced by isolate N2215 with concentration 16.48 ppm. From these results, it proved that *F. verticillioides* and *F. proliferatum* isolates are capable of producing a high level of FB_1_. However, there were six isolates (three isolates from each species) that were not capable of producing FB_1_ and no detection from UFLC analysis.

**Figure 1:**
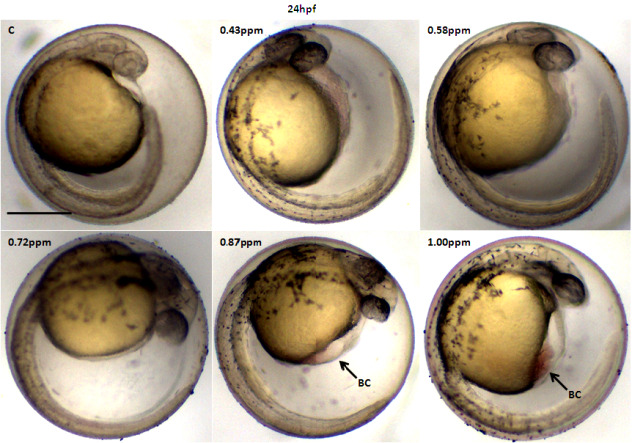
Representative optical images of deformed zebrafish larvae at 24 hpf caused by FB_1_ exposure. Malformations are indicated by arrows. BC: blood clotting. Scale bar: 500 μm.

**Figure 2:**
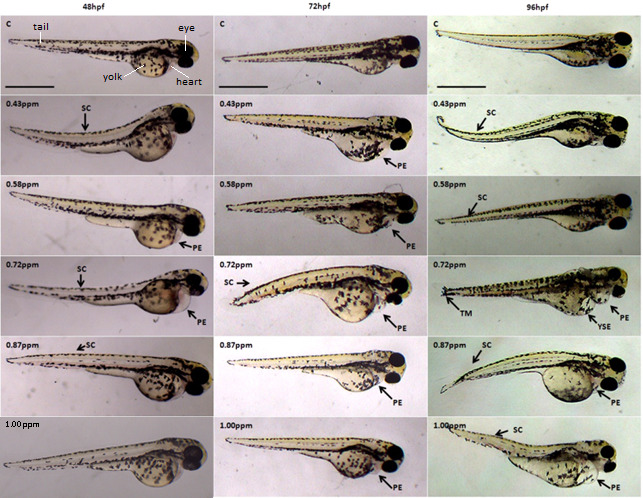
Representative optical images of deformed zebrafish larvae at 48 hpf, 72 hpf and 96 hpf caused by FB_1_ exposure. Malformations are indicated by arrows. SC: spinal curvature; TM: tail malformation; PE: pericardial edema; YSE: yolk sac edema; BC: blood clotting. Scale bar: 500 μm.

**Figure 3:**
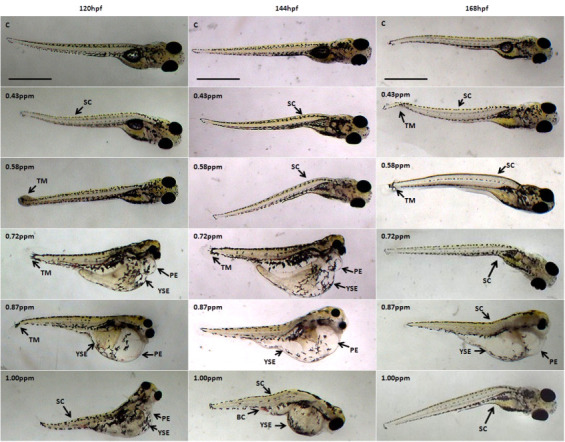
Representative optical images of deformed zebrafish larvae at 120 hpf, 144 hpf and 168 hpf caused by FB_1_ exposure. Malformations are indicated by arrows. SC: spinal curvature; TM: tail malformation; PE: pericardial edema; YSE: yolk sac edema. Scale bar: 500 μm.

The effects of FB_1_ exposure toward zebrafish larvae can be seen as early as 24 hpf where blood clotting was formed at 0.87 ppm and 1.00 ppm (Fig. 1). From the control larvae and concentration lower than 0.72 ppm, no significant morphological abnormality was observed. However, starting at 48 hpf, more abnormalities development can be seen from exposure at the lowest concentration of 0.43 ppm, the zebrafish showed spinal curvature and also pericardial edema was observed at 0.58 ppm and 0.72 ppm of FB_1_ exposure. The severity of pericardial edema increases at 72 hpf onwards with exposure 0.72 ppm, 0.87 ppm and 1.00 ppm (Figs. 2 and 3).

### Tail Coiling, Heartbeat and Mortality Rate of Larval Zebrafish

Tail coiling rate of zebrafish reduced significantly as the concentration of the FB_1_ increased. Control zebrafish showed the highest rate of tail coiling which was 3.63 ± 1.58 per min and had a significant difference compared to other concentrations (Table 2). Meanwhile the tail-coiling rate of the highest FB_1_ concentration, 0.87 ppm and 1.00 ppm had no significant difference.

Heartbeat rate that was measured at 48 hpf showed no significant difference at all different concentrations. A normal heartbeat rate of zebrafish is at 120–180 beats per min (bpm). As shown in Table 2, the range of heart rate of control and treated zebrafish was from 144.33 bpm–161.29 bpm, which was still under normal range. At 48 hpf, heart morphology only showed mild abnormalities and mild pericardial edema.

**Figure 4:**
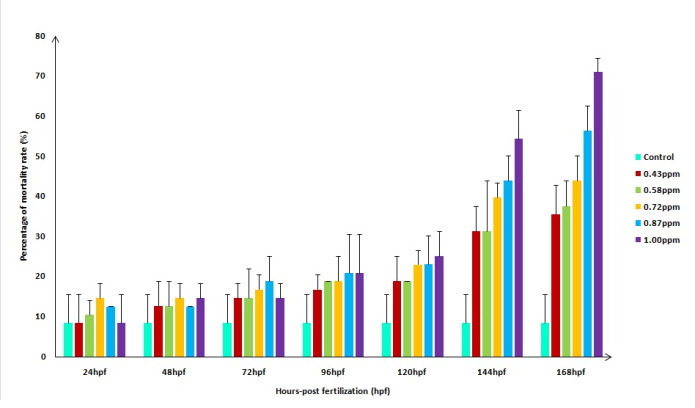
Mortality rate of zebrafish larvae exposed with different concentrations of FB_1_ according to hours post-fertilisation (hpf).

Fig. 4 shows the mortality rate of zebrafish larvae that were exposed to FB_1_ according to hpf. For the control treatment, the mortality rate at 24 hpf was 8.33% and there were no significant changes until 168 hpf. The mortality happened at control treatment were due to unfertilised eggs. For all FB_1_ treatment, the mortality rate started showing significant changes at 144 hpf. Zebrafish larvae that were exposed to 0.43 ppm of FB_1_ had 18.77% of the mortality rate at 120 hpf and increased significantly to 31.27% of mortality rate at 144 hpf. The highest concentration of FB_1_ was 1.00 ppm and the mortality rate at 120 hpf was 25.03%, increased significantly to 54.17% at 144 hpf and rising to 70.87% at 168 hpf.

**Figure 5:**
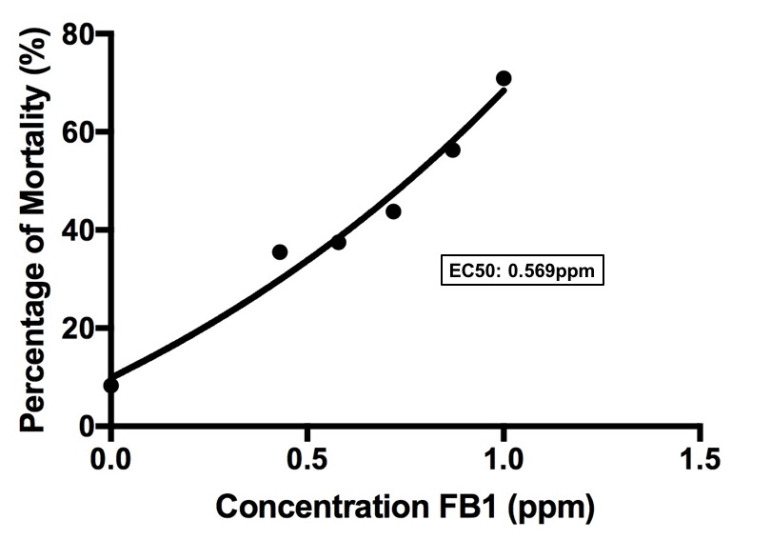
Concentration-response curve of FB_1_ towards zebrafish.

To determine the mortality effect of FB_1_ towards zebrafish, effective concentration (EC_50_) was calculated based on concentration-response curve (Fig. 5). Based on the curve, EC_50_ of FB_1_ towards zebrafish was 0.569 ppm.

## DISCUSION

### Amplification of *FUM1* in *F. proliferatum* and *F. verticillioides*

Out of 48 isolates were used in *FUM1* gene detection study, 21 isolates were *F. verticillioides* and 27 isolates were *F. proliferatum*. This result can be compared with previous research (Nor Azliza *et al*. 2017) detected 780 bp of *FUM1* gene in 11 isolates of *F. verticillioides*, 49 isolates of *F. proliferatum*, 24 isolates of *F. fujikuroi* and 10 isolates of *F. oxysporum* from various range of host plants. Another report by Dissanayake *et al*. (2009) found that 13 out of 20 isolates of *F. proliferatum* isolated from Welsh onion have successfully amplified 780 bp of *FUM1* gene specific fragments.

The absence of *FUM1* gene in two isolates of *F. proliferatum* (B68 and B92) might be caused by mutation where there were defects in *FUM1* gene expression or might be due to the movement of gene cluster within genome during the evolution of the species (Proctor *et al*. 2004). It is important to identify the presence of *FUM1* gene before quantifying the level of FB_1_ produce in order to see the correlation between the presences of the gene and the level of the mycotoxin produced.

### FB_1_ Production using UFLC Analysis

There were six isolates (three isolates from each species) that were not capable of producing FB_1_ and no detection from UFLC analysis. Even though *FUM1* gene was amplified in *Fusarium* isolates, it does not reflect the FB_1_ production. The biosynthesis of FB_1_ also can be influenced and altered by the changes in abiotic factors. The average concentration of FB_1_ produced by *F. verticillioides* and *F. proliferatum* was 143.99 ppm and 655.92 ppm respectively which is lower than previous studies (Choi *et al*. 2018) where *F. verticillioides* produced 0.6–4749.1 μg g^−1^ (ppm) FB^1^ and *F. proliferatum* produced 0.9–7969.0 μg g^−1^ (ppm) FB_1_. Meanwhile another research by Lee *et al*. (2012) has reported that *F. verticillioides* produced 2686.4 μg g^−1^ (ppm) FB_1_ and *F. proliferatum* produced 974.0 μg g^−1^ (ppm) FB_1_. High water activity will lead to higher fungal growth thus more fumonisin will produce (Samapundo *et al*. 2005). Besides that, the C:N ratio also can influence the production of fumonisin by activating selective genes that are outside of the *FUM* gene cluster. For example, when C source is increasing, *ZFR1* and *FST1* genes will regulate the FB_1_ biosynthesis (Jimenez *et al*. 2003; Jurado *et al*. 2008). Meanwhile, when the N source is decreasing, *FUM1, FUM8* and *AREA* genes will significantly increase and induce the production of fumonisin (Kohut *et al*. 2009).

### Morphological Abnormalities of Zebrafish Larvae Exposed to FB_1_

The severity of pericardial edema increases at 72 hpf onwards with exposure 0.72 ppm, 0.87 ppm and 1.00 ppm (Figs. 2 and 3). Since currently there is no evidence and reports of FB_1_ exposure on zebrafish, all the comparisons can only be made with other studies of different type mycotoxins and chemicals exposure on zebrafish. Pericardial edema is one of the signs of kidney failure where the fluid accumulated in the body cavity and causing swollen of the heart (Hanke *et al*. 2013). The decrease in plasma oncotic pressure causes the excess fluid buildup indicates failure of the kidney where the pronephric tubules were unable to reabsorb the essential protein (Kemp & Conte 2012; Hanke *et al*. 2013). This is significant to a human disease called proteinuria where there are excess proteins released through urine.

Severe yolk sac edema can be observed at 120 hpf with exposure of 0.72 ppm and higher. The previous report described the effect of zebrafish larvae that were exposed to thifluzamide fungicide also showed yolk sac edema and pericardial edema (Yang *et al*. 2016). Yolk sac edema also was one indication of kidney failure that involved the osmotic pressure. Kidney failure can cause permeability defects and ionic imbalance thus lead to edema and creating a positive feedback loop. The edema later will disrupt the function of kidney and circulatory function and further worsen the edema. This positive feedback loop of edema is irreversible (Hill *et al*. 2004).

Spinal curvature can be seen at all concentrations as early as at 48 hpf. The spine can be observed either curve upward, curve downward or appear undulate. Yin *et al*. (2015) stated that the exposure of fusaric acid towards zebrafish have caused all the embryos to develop undulated spine when treated with 400 μM–600 μM (72 ppm–108 ppm) of fusaric acid. However, no report on when expose to FB_1_.

### Tail Coiling, Heartbeat and Mortality Rate of Larval Zebrafish

Spontaneous tail coiling was reported as the first behaviour of zebrafish showing the development of the motor activity. Tail coiling is independent of sensory stimulation starts at 17 hpf and gradually decreasing at 26 hpf (Brustein *et al*. 2003). During coiling, the tail movement was non-myogenic and originates from spinal cord, primary motoneurons and a restricted number of interneurons (Hirata *et al*. 2005). Fumonisin exposure might have caused interference on the development of the nervous system as it significantly reduced the tail-coiling rate of zebrafish.

The effect of FB_1_ on heart just started and did not interrupt the heart rate thus revealed the non-significance difference of heart rate. Exposure of pyridinebased pesticide (PPF) on zebrafish embryos and the effects of heart rate showed that there was no significant difference in the heart rate in 0.16 and 0.33 μg mL^−1^ till 96 hpf (Maharajan *et al*. 2018). However, embryos that were exposed to higher concentration of 1.66 μg mL^−1^ at 48 and 72 hpf significantly increase the heart rate.

From the result, it showed that the effect of FB_1_ on mortality of zebrafish larvae was late at day 6 (144 hpf) of exposure and increasing as exposure time increased. The mortality also happened due to several abnormalities and malfunction of the system in zebrafish, for example, circulatory and excretory systems. Exposure of 50 μM of citrinin at 72 hpf showed viability of 71.6% ± 10.5% and reduced completely to 0% at 120 hpf where none of the zebrafish survived (Wu *et al*. 2013). The study also stated that the viability of zebrafish decreases as the concentration and time of exposure to citrinin increased. T-2 toxin exposure to zebrafish larvae have found that 0.80 μmol L^−1^ caused 100% mortality at 24 hpf. Meanwhile, zebrafish treated with 0.20 μmol L^−1^, 0.25 μmol L^−1^, 0.30 μmol L^−1^ and 0.40 μmol L^−1^ have caused 0%, 5.5%, 30.5% and 83.3% mortality rate respectively at 24 hpf (Yuan *et al*. 2014). These results indicated that FB_1_, citrinin and T-2 toxin have different levels of toxicity towards zebrafish depending on the concentration level and time of exposures. Since this is the first report of exposure of FB_1_ on zebrafish larvae, higher concentrations should be applied on the larvae as well as increasing the time of exposures to get the 100% mortality rate. Based on this study, EC_50_ of FB_1_ towards zebrafish was 0.569 ppm. The previous report found that the EC_50_ of T-2 toxin towards zebrafish was 0.18 μmol L^−1^ (0.084 ppm) (Yuan *et al*. 2014). The value is lower as compared to FB_1_ and it indicates that FB_1_ requires higher concentration for the effective response of 50% mortality.

## CONCLUSION

Detection of *FUM1* gene on *F. proliferatum* and *F. verticillioides* revealed that 48 out of 50 isolates gave positive amplification of *FUM1* gene and from quantification of FB_1_ by using UFLC, 18 out of 21 isolates of *F. verticillioides* produced FB_1_ ranging from 12.26 ppm–954.01 ppm. Meanwhile, 26 out of 29 isolates of *F. proliferatum* produced FB_1_ ranging from 16.48 ppm–6677.32 ppm. Embryotoxicity test of FB_1_ on zebrafish showing significant effects on the mortality rate and morphological abnormalities. Meanwhile the EC_50_ of FB_1_ towards zebrafish was 0.569 ppm. In addition, knowledge on the FB_1_ effects on larval zebrafish will help to understand further the pathology of associated acute and chronic diseases in an aquatic animal.

## Figures and Tables

**Table 1:** Concentrations of FB_1_ produced by *F. verticillioides* and *F. proliferatum.*

No.	Species	Isolate No.	Host	Locality	Concentration (ppm)
1	*F. verticillioides*	A2358	*Musa acuminata*	Hutan Melintang, Perak	25.03
2		A2359	*Musa acuminata*	Hutan Melintang Perak	87.17
3		B106	*Zea mays*	Serdang, Selangor	ND
4		B146	*Zea mays*	Serdang, Selangor	71.93
5	*F. verticillioides*	B1371	*Zea mays*	Semenyih, Selangor	ND
6		C116	*Zea mays*	Cameron Highland, Pahang	53.91
7		C121	*Zea mays*	Cameron Highland, Pahang	47.46
8		J44	*Zea mays*	Senggarang, Johor	268.07
9		J1361	*Zea mays*	Sri Medan, Johor	91.49
10		J1362	*Zea mays*	Sri Medan, Johor	77.80
11		J1363	*Zea mays*	Senggarang, Johor	954.01
12		J1364	*Zea mays*	Senggarang, Johor	227.53
13		K2344	*Musa acuminata*	Bukit Kayu Hitam, Kedah	158.86
14		N1387	*Zea mays*	Rembau, Negeri Sembilan	66.72
15		P1367	*Zea mays*	Seberang Prai, Pulau Pinang	88.65
16		7696	*Zingiber officinale*	Gelugor, Pulau Pinang	12.26
17		7697	*Zingiber officinale*	Gelugor, Pulau Pinang	99.60
18		7703	*Zingiber officinale*	Batu Uban, Pulau Pinang	67.23
19		7704	*Zingiber officinale*	Gelugor, Pulau Pinang	159.79
20		7705	*Zingiber officinale*	Gelugor, Pulau Pinang	34.27
21		7706	*Zingiber officinale*	Batu Uban, Pulau Pinang	ND
22	*F. proliferatum*	B68	*Zea mays*	Serdang, Selangor	ND
23		B92	*Zea mays*	Serdang, Selangor	ND
24		B1777	*Luffa acutangula*	Tanjung Karang, Selangor	96.73
25		B1778	*Luffa acutangula*	Tanjung Karang, Selangor	991.06
26		B1779	*Luffa acutangula*	Tanjung Karang, Selangor	524.74
27		B1780	*Luffa acutangula*	Tanjung Karang, Selangor	415.02
28		B1781	*Luffa acutangula*	Tanjung Karang, Selangor	152.14
29		B1784	*Luffa acutangula*	Tanjung Karang, Selangor	227.34
30		B2377	*Musa acuminata*	Tanjung Karang Selangor	189.98
31		B2433	*Musa acuminata*	Serdang Selangor	6677.32
32		F286	*Cosmos caudatus*	Puchong, Selangor	31.20
33		J1789	*Curcubita pepo*	Tangkak, Johor	188.17
34		J1790	*Curcubita pepo*	Tangkak, Johor	731.54
35		J1791	*Curcubita pepo*	Tangkak, Johor	114.89
36		J1792	*Curcubita pepo*	Tangkak, Johor	616.52
37		J1793	*Curcubita pepo*	Tangkak, Johor	194.86
38		M2396	*Musa balbisiana*	Masjid Tanah, Melaka	3956.23
39		M2399	*Musa paradisiaca*	Merlimau, Melaka	311.80
40		N2215	*Zea mays*	Rembau, Negeri Sembilan	16.48
41		P202	*Zea mays*	Seberang Prai, Pulau Pinang	ND
42		P204	*Zea mays*	Seberang Prai, Pulau Pinang	154.01
43		P1366	*Zea mays*	Seberang Prai, Pulau Pinang	103.79
44	*F. proliferatum*	680	*Oryzae sativa*	Haji Kudung, Kedah	123.75
45		901	*Asparagus officinalis*	Kundasang, Sabah	579.02
46		971	*Triticum aestivum*	Teluk Kumbar, Pulau Pinang	229.52
47		1007	*Sorghum bicolor*	Sri Aman, Sarawak	48.95
48		1380	*Dendrobium* sp.	Kuala Lumpur	319.27
49		3240	*Saccharum officinarum*	Padang Terap, Kedah	17.74
50		3244	*Saccharum officinarum*	Padang Terap, Kedah	41.80

ND: not detected

**Table 2:** Effect of FB_1_ on tail coiling rate and heartbeat rate of *Danio rerio*.

Concentration (ppm)	Tail coiling rate (per min)	Heart rate (beats per min)
0	3.63 ± 1.58^a^	161.29 ± 49.52^a^
0.43	2.73 ± 1.20^b^	152.79 ± 58.96^a^
0.58	2.50 ± 1.20^bc^	149.23 ± 57.49^a^
0.72	2.30 ± 1.24^bc^	145.10 ± 61.35^a^
0.87	2.13 ± 1.06^c^	145.88 ± 56.42^a^
1.00	2.02 ± 0.86^c^	144.33 ± 61.04^a^

*Note*: Values are mean and standard deviation from three replicates. Tail coiling and heartbeat rate were counted within 1 min at 24 hpf and 48 hpf, respectively for every larva according to OECD (2012) guideline. Superscripts on each column with different letters indicate significant difference among means (Duncan test, *p* < 0.05).
